# Deficits in Auditory and Visual Sensory Discrimination Reflect a Genetic Liability for Psychosis and Predict Disruptions in Global Cognitive Functioning

**DOI:** 10.3389/fpsyt.2020.00638

**Published:** 2020-07-07

**Authors:** Ian S. Ramsay, Michael-Paul Schallmo, Bruno Biagianti, Melissa Fisher, Sophia Vinogradov, Scott R. Sponheim

**Affiliations:** ^1^ Department of Psychiatry and Behavioral Sciences, University of Minnesota, Minneapolis, MN, United States; ^2^ Department of R&D, Posit Science Corporation, San Francisco, CA, United States; ^3^ Minneapolis Veterans Affairs Medical Center, Minneapolis, MN, United States

**Keywords:** psychosis, sensory discrimination, auditory perception, visual perception, endophenotype, global cognition

## Abstract

Sensory discrimination thresholds (i.e., the briefest stimulus that can be accurately perceived) can be measured using tablet-based auditory and visual sweep paradigms. These basic sensory functions have been found to be diminished in patients with psychosis. However, the extent to which worse sensory discrimination characterizes genetic liability for psychosis, and whether it is related to clinical symptomatology and community functioning remains unknown. In the current study we compared patients with psychosis (PSY; N=76), their first-degree biological relatives (REL; N=44), and groups of healthy controls (CON; N=13 auditory and visual/N=275 auditory/N=267 visual) on measures of auditory and visual sensory discrimination, and examined relationships with a battery of symptom, cognitive, and functioning measures. Sound sweep thresholds differed among the PSY, REL, and CON groups, driven by higher thresholds in the PSY compared to CON group, with the REL group showing intermediate thresholds. Visual thresholds also differed among the three groups, driven by higher thresholds in the REL versus CON group, and no significant differences between the REL and PSY groups. Across groups and among patients, higher thresholds (poorer discrimination) for both sound and visual sweeps strongly correlated with lower global cognitive scores. We conclude that low-level auditory and visual sensory discrimination deficits in psychosis may reflect genetic liability for psychotic illness. Critically, these deficits relate to global cognitive disruptions that are a hallmark of psychotic illnesses such as schizophrenia.

## Introduction

Disruptions in early sensory processing are prominent in psychosis-spectrum disorders (1, 2), and are hypothesized to underlie the clinical features of illnesses such as schizophrenia and bipolar disorder. Impairments in the integration of low-level sensory signals have been found to be a likely contributor to the neural pathophysiology observed in psychosis (3), as well as related to psychotic symptomatology (4–6) and cognitive deficits (7). Electrophysiological responses for both auditory and visual stimuli have consistently demonstrated impairments in sensory processing in patients with psychosis. For example, patients with schizophrenia show impaired passive and volitional attention in response to static auditory stimuli reflected in reduced auditory mismatch negativity (MMN) (8) and diminished P300 and N100 responses during an auditory oddball task (9, 10). Patients with schizophrenia also demonstrate deficits in perception of dynamic auditory stimuli, as evidenced by impaired discrimination of both temporally modulated and unmodulated tones (5). Individuals with schizophrenia also have well known visual processing deficits as evidenced by abnormal visual evoked potentials (11–16).

Bipolar disorder patients have also shown auditory MMN deficits (17), as well as visual sensory processing deficits indexed by altered visual MMN/P300 (18), diminished P100 response on a “Go-No-Go” task (19), and diminished P300 on a contour perception task (20). Across psychotic disorders, these deficits appear to have downstream consequences for ‘higher-order’ cognition such as memory and executive control, and contribute to global cognitive impairments observed in these populations (1). Sensory disruptions have also been shown to contribute to poor functional outcomes in these populations (21, 22), suggesting that early sensory processing is a critical treatment target.

Sensory discrimination thresholds refer to the level or intensity at which a stimulus can be reliably perceived. Recent efforts to behaviorally assess sensory discrimination of auditory and visual stimuli have relied on auditory and visual sweep paradigms. “Sound Sweeps” is an auditory frequency discrimination time-order judgement task wherein participants are asked to determine whether a pair of frequency-modulated tones are increasing or decreasing in frequency. Similarly, “Visual Sweeps” is a visual frequency discrimination time-order judgement task that requires the participant to determine whether a Gabor patch is modulating inward or outward. Both tasks use a staircase function to increase or decrease the duration of each stimulus, thereby adjusting the task’s difficulty and allowing for an adaptive and accurate assessment of an individual’s speeded auditory or visual sensory discrimination threshold. Developed by Posit Science Inc., these tasks can be delivered via a computer or a tablet, making them deployable at a larger scale than more traditional psychophysics tasks (i.e. in an outpatient clinic or remotely). Both auditory and visual thresholds have been previously shown to be impaired in schizophrenia (23), and reflect functional outcomes such as work performance or community functioning (21, 22).

Sound Sweeps have been studied extensively in prior work, with a focus on using this task as a part of targeted cognitive training (TCT) of the auditory system. TCT programs utilizing Sound Sweeps have been shown to enhance cognitive functioning in early (24), chronic (25), and treatment refractory schizophrenia (26). TCT has also demonstrated structural (27), functional (28, 29), electrophysiological (30, 31), and oscillatory (32) plasticity in these populations. Notably, target engagement of the auditory system indexed by Sound Sweeps was shown to mediate global cognitive improvements from TCT (33), and may therefore be an early marker of response to the treatment. This has also been established by studies demonstrating that a brief (1 h) course of Sound Sweeps is predictive of subsequent treatment gains in response to TCT (34, 35).

Previous work demonstrated a relationship between auditory sensory discrimination measured using the Sound Sweeps paradigm and measures of auditory working memory, attention, verbal memory, and executive functioning in a group of outpatient schizophrenia patients (36). These findings align with electrophysiological work demonstrating that early auditory processing plays an important role in cognitive and psychosocial functioning in schizophrenia (37). But what remains unknown is the extent to which visual sensory threshold disruptions may also reflect aspects of global cognitive dysfunction in this population. Additionally, given evidence of early sensory/attention processes being associated with a genetic liability for psychosis (38), the extent to which sensory discrimination thresholds also reflect this genetic liability is not known.

The current study used the Sound and Visual Sweeps paradigms to examine sensory discrimination in a group of patients with psychosis (PSY), their first-degree relatives (REL), and a sample of controls (CON) drawn from the community. We hypothesized that both auditory and visual sensory processing deficits would reflect a genetic liability for psychosis, where relatives as a group would occupy an intermediate deficit in sensory thresholds given varying levels of genetic liability for the disorder. We also investigated the extent to which auditory and visual discrimination thresholds correspond to psychotic symptomology, global cognitive measures, and role and social functioning.

## Methods

### Participants

Participants were recruited from three separate sources:

- Psychosis Human Connectome Project (pHCP): 78 outpatient participants with a primary psychotic (PSY) disorder (Schizophrenia N=25, Schizoaffective Disorder N=24, Bipolar Disorder I N=24, Psychosis NOS N=2), 44 first-degree biological relatives (REL) of the psychosis subjects (N=15 with a previous mood disorder diagnosis, N=2 with a previous substance use disorder diagnosis, N=2 with a previous psychotic-spectrum diagnosis, N=1 with a previous anxiety disorder diagnosis), and 13 healthy controls (CON) were all recruited and screened using the Structured Clinical Interview for DSM-V (SCID). All subjects completed both Sound and Visual Sweeps, in addition to assessment of symptoms, cognition, and functioning. Subjects were excluded for alcohol or drug abuse in the past month, and alcohol or drug dependence in the last 6 months. Subjects were also excluded if they had an estimated IQ<70, or had compromised vision or hearing (i.e. legally blind or unable to hear without a hearing aid).
- Minnesota State Fair (‘The Great Minnesota Get-Together’): 275 community control participants completed Sound Sweeps as part of a larger battery of assessments administered at the Minnesota State Fair.
- Amazon’s Mechanical Turk (M-Turk): 278 subjects remotely completed the Visual Sweeps task as part of a larger study performed by Biagianti and colleagues (23).

All study procedures were approved by the Institutional Review Board of the University of Minnesota, and all participants gave written informed consent.

### Assessment Procedures

#### Sound and Visual Sweeps Paradigms

**Figure 1:**
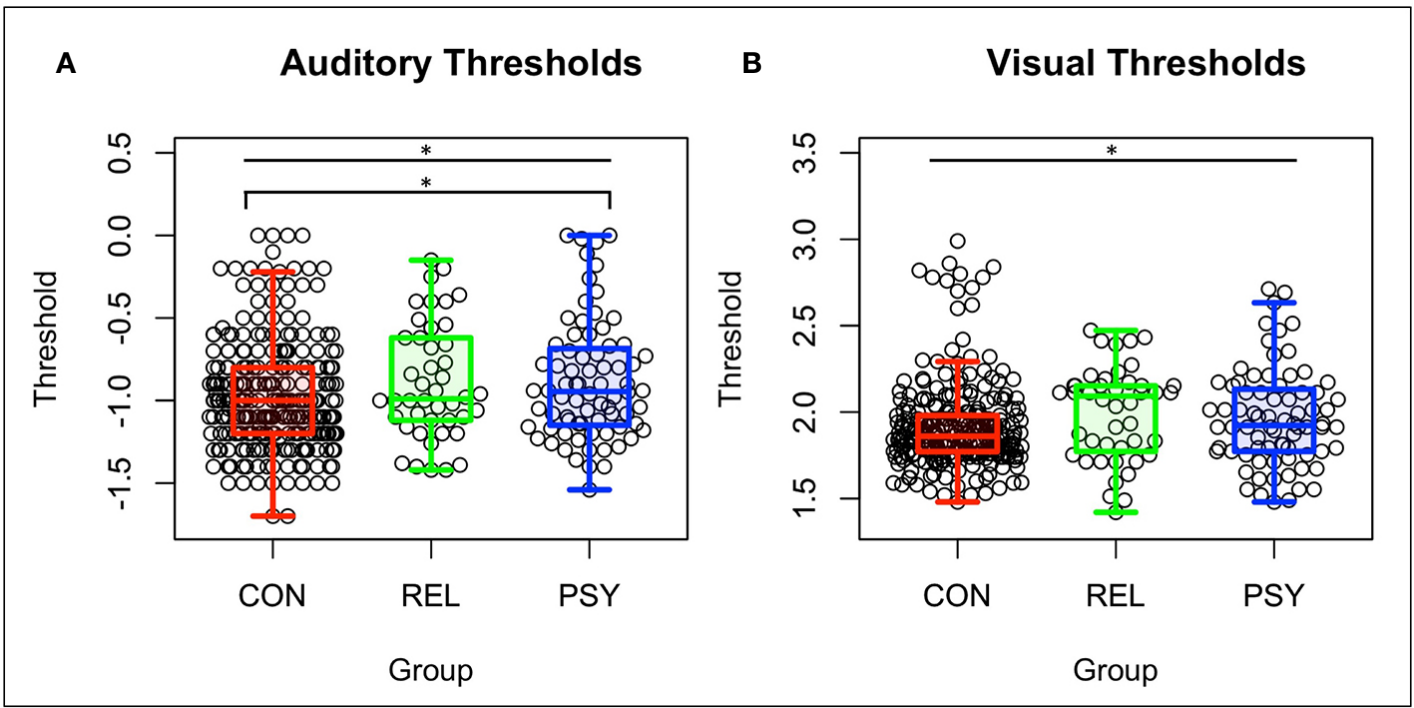
Auditory and visual thresholds. Note: Lower thresholds indicate better performance. **(A)** Auditory thresholds (log10 of ISI x 1,000) from the Sound Sweeps task were found to be different between the CON, REL, and PSY groups (F=4.32 p=.01), driven by lower thresholds in CON vs PSY (p=.037). **(B)** Visual thresholds (log10 of ISI) from the Visual Sweeps task were found to be different between CON, REL, and PSY groups (F=3.90 p=.02), driven primarily by marginally lower thresholds in CON vs REL (p=.05). CON, Controls; REL, Relatives; PSY, Psychosis; ISI, Inter-stimulus interval. *=p<.05.

As described in Biagianti et al., (23) (see also Figure 1 in (25)), Sound Sweeps is a frequency time-order judgement task that indexes auditory psychophysical speed and efficiency. Participants are presented with a sequence of two tones, and are asked to determine whether the frequency modulation of each tone goes from a higher to a lower pitch, or a lower to a higher pitch. Using a 2-down-1-up staircase procedure, two correct responses will adaptively increase the difficulty by shortening the sweep duration and interstimulus interval (ISI; which are held equal), while 1 incorrect response will lengthen the sweep duration/ISI. The sweep duration starts at 251 ms, with a minimum of 31 ms, and a maximum of 1,000 ms. The staircase function terminates after 40 trials, ending the task. Auditory discrimination threshold is calculated by the logarithm with base 10 of the number of seconds of the ISI (in ms) divided by 1,000 (to ensure normally distributed data); this threshold is expected to converge on the ISI for which the participant correctly responded on 70.7% of trials (39). Therefore, lower thresholds reflect more sensitive discrimination ability and enhanced processing of brief auditory stimuli.

Visual Sweeps operates similarly in that it is also a frequency time-order judgement task, but indexes visual perceptual discrimination and attention. Participants are presented with two successive visual Gabor patches, and are asked to determine whether the spatial frequency modulation is moving from higher to lower (“outward”), or from lower to higher (“inward”). This task also operates using a 2-down-1-up staircase procedure, where 2 successive correct responses will adaptively decrease the sweep duration and ISI, and 1 incorrect response will lengthen the duration/ISI. The sweep duration starts at 200 ms, with a minimum of 10 ms, and a maximum of 1,000 ms. This task also terminates after 40 trials ending the task. The visual threshold is calculated using the logarithm with base 10 of the number of seconds of the ISI (in ms) at which the participant correctly responded on approximately 70.7% of trials.

In the pHCP and Minnesota State Fair samples, all participants performed both tasks on an Apple iPad using the Safari web browser from a comfortable viewing distance. The State Fair participants were given headphones to complete the task, while the pHCP subjects completed the tasks without headphones in a quiet room. The M-Turk participants completed the task on a personal device of their choosing. We could not ascertain whether individuals were using headphones or not, but only subjects who were determined to have adequately engaged in the task were included in the final analyses (see below).

#### Symptoms, Cognitive, and Functional Assessments

Participants from the pHCP sample underwent additional assessments of symptoms, cognition, and functioning. Positive, negative, and global psychiatric symptoms were measured using the Scale for Assessment of Positive Symptoms (SAPS) (40), the Scale for Assessment of Negative Symptoms (SANS) (41), and the Brief Psychiatric Rating Scale (BPRS) (42). Cognition was assessed using the Brief Assessment of Cognition in Schizophrenia (BACS) (43, 44). The BACS contains subtests measuring verbal learning and memory, working memory, verbal fluency, motor speed, processing speed, and problem solving, all of which are age and gender-normed to derive a ‘global cognition’ score. Functioning was measured using the Global Assessment of Functioning Role and Social Scales (45).

### Planned Analyses

Group analyses compared PSY, REL, and CON using subjects from the pHCP and State Fair for Sound Sweeps, and pHCP and Mechanical Turk for Visual Sweeps. Subjects whose auditory or visual threshold was 3 *SD*s greater than the mean were removed, as these subjects likely did not understand or adequately engage in the task (2 PSY from the pHCP dataset, 0 REL, and 12 CON subjects from the M-Turk data set were removed). Next, ANCOVAs controlling for age and gender were performed for Sound and Visual Sweeps separately. These were then followed by Tukey’s HSD test to identify which groups may be driving an effect. Next we used linear models, again controlling for age and gender, to examine the relationships between auditory and visual thresholds and measures of symptoms, cognition, and functioning. We used a false-discovery rate (FDR) correction to account for multiple comparisons. Last, we followed up these analyses by performing within group correlations in the pHCP sample to determine the strengths of these relationships within the PSY, REL, and CON groups (this left N=13 in the CON group for these analyses), and also followed up on correlations between the PSY group and chlorpromazine equivalents (CPZ) to assess whether there were any relevant effects of medication.

## Results

The PSY, REL, and CON groups differed on the basis of age (RELs were older), gender distribution (CON had more females), medication dosage, symptom severity measured by the SANS, SAPS, and BPRS, global cognition measured by BACS (PSY and REL showed deficits compared to CON), and both role and social global functioning (PSY showed lower global functioning; See **Table 1**). Auditory thresholds measured by the Sound Sweeps task (controlling for age and gender) differed between groups (*F*(**2**,**394**)=4.32; *p*=.01; See **Figure 1A**), driven by lower thresholds in the CON compared to PSY group (*Tukey’s HSD* *p*=.035), while the REL group showed lower but not significantly different thresholds from CON (REL vs. CON: *Tukey’s HSDp*=.13) and no differences from PSY (*Tukey’s HSD* *p*=.99). Visual thresholds measured by the Visual Sweeps task (controlling for age and gender) also differed between groups (*F*=3.90(2,383); *p*=.02; See **Figure 1B**), characterized by lower but not significantly different thresholds in CON versus PSY (*Tukey’s HSDp*=.14), a similar effect in the CON versus REL groups (*Tukey’s HSD* *p*=.053), and no differences between the PSY and REL groups (*Tukey’s HSDp*=.75). Within our 3 sub-groups of control subjects, neither auditory or visual thresholds differed between the pHCP and State Fair or M-Turk samples respectively (*p’s>*.19). Within the PSY group, we followed up on whether specific diagnosis affected sensory thresholds. Neither Sound (F(3,69)=2.26; p=.09; this trend was driven by higher thresholds in N=2 subjects with Psychosis Not Otherwise Specified) nor Visual Sweeps threshold (F(3,69)=.40; p=.78) differed on the basis of psychiatric diagnosis. These results indicate that auditory and visual discrimination performance are similar across dimensions of psychosis (e.g., among those with or without significant mood symptoms).

**Table 1:** Demographics.

	Psychosis (N=76)	Relatives (N=44)	Controls (pHCP; N=13)	Controls (MN State Fair/Sound Sweeps; N=275)	Controls (M-Turk/Visual Sweeps; N=267)	F-value/T-value/Chi-Squared	p-value
Age	M=35.88; SD=12.83	M=42.90 SD=14.27	M=48 SD=8.94	M=33.79 SD=18.02	M=27.99 SD=8.99	14.22	0.0000009
Gender	Male=41 Female=34	Male=15 Female=26	Male=3 Female=10	Male=88 Female=182	Male=145 Female=122	4.4	0.04
CPZ	M=4.66 SD=5.54 (N=61)	M=1.49 SD=4.22 (N=10)	–	–	–	2.10	.05
SANS	M=27.89 SD=18.58	–	–	–	–	–	–
SAPS	M=14.67 SD=14.71	–	–	–	–	–	–
BPRS	M=45.26 SD=11.49	M=31.88 SD=7.29	M=29.75 SD=4.83	–	–	30.44	1.82E-11
BAC Global Score (Z)	M=-.62 SD=1.23	M=-.12 SD=1.24	M=.45 SD=.69	–	–	5.33	0.006
GAF Role	M=4.54 SD=2.79	M=7.08 SD=2.75	M=8.25 SD=1.22	–	–	17.5	0.0000002
GAF Social	M=5.72 SD=2.12	M=6.78 SD=2.53	M=7.92 SD=.9	–	–	6.75	0.002

Demographics for the Psychosis, Relatives, and Control groups. CPZ, Chlorpromazine Equivalents; SANS, Scale for the Assessment of Negative Symptoms (Range: 0–125); SAPS, Scale for the Assessment of Positive Symptoms (Range: 0–170); BPRS, Brief Psychiatric Rating Scale (Range: 24–168); BAC, Brief Assessment of Cognition; GAF Role, Global Assessment of Functioning: Role (Range: 1–10); GAF Social, Global Assessment of Functioning: Social (Range: 1–10).

**Figure 2:**
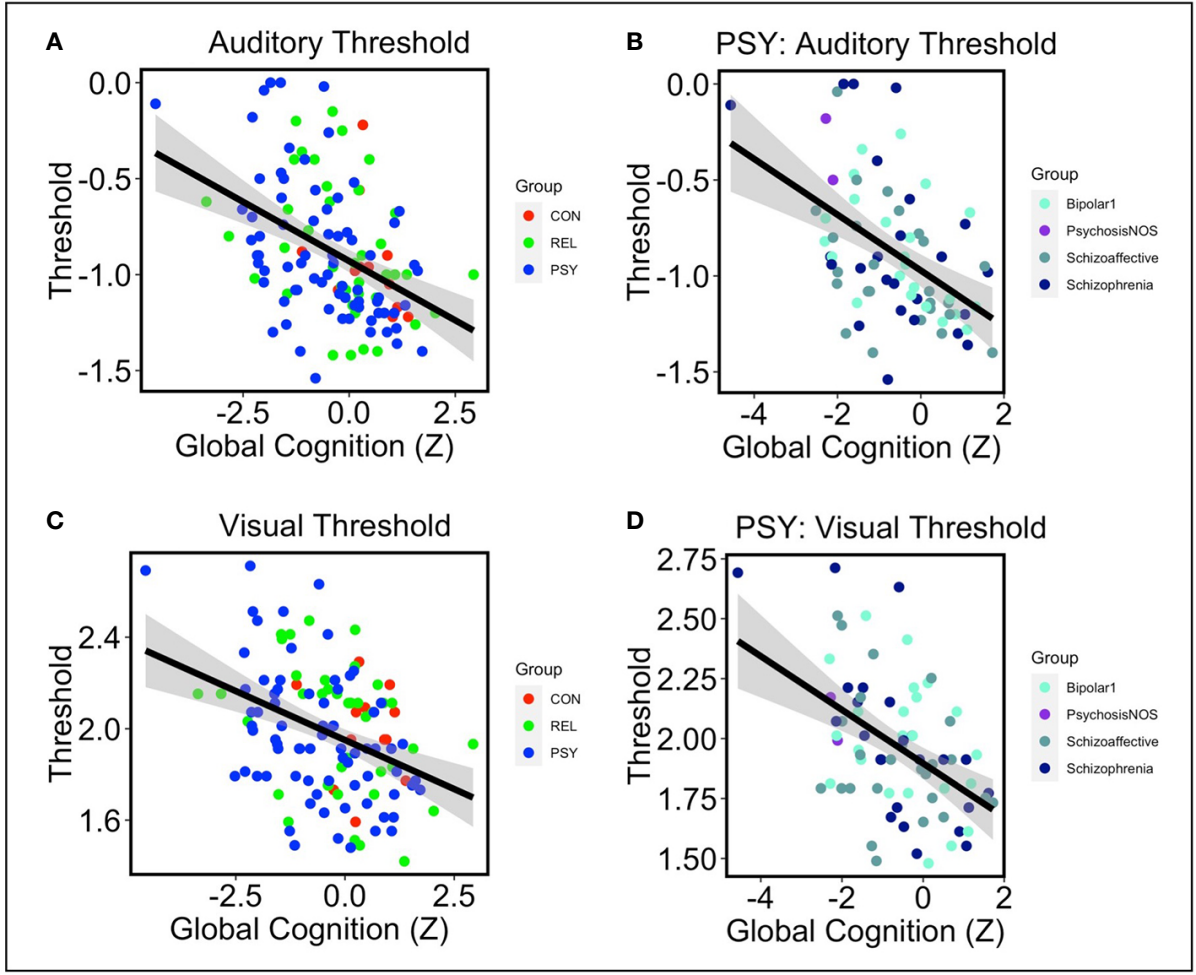
Note: **(A)** Lower auditory thresholds (log10 of ISI x 1,000) were associated with higher global cognition scores measured by the BACS across CON (Red), REL (Green), and PSY (Blue) groups controlling for age and gender (t=-4.83 p=.000004). **(B)** Within the PSY group, lower auditory thresholds correlated with higher cognition scores (r=-.49 p=.00001). This was also the case when examining individual DSM-V diagnoses: Bipolar Disorder 1 (r=-.46 p=.03), Psychosis NOS (not examined as N=2), Schizoaffective Disorder (r=-.46 p=.02), Schizophrenia (r=-.48 p=.02). **(C)** Lower visual thresholds (log10 of ISI) were also associated with higher global cognition across CON, REL, and PSY groups controlling for age and gender (t=-3.2 p=.002). **(D)** Within the PSY group, lower visual thresholds correlated with higher cognition scores (r=-.48 p=.00001). This was primarily driven by the subjects with a Schizophrenia diagnosis (r=-.70 p=.0001) and non-significant effects in the Bipolar Disorder 1 (r=-.36 p=.11) and Schizoaffective Disorder groups (r=-.30 p=.14). CON, Controls; REL, Relatives; PSY, Psychosis; BACS, Brief Assessment of Cognition in Schizophrenia.

Next we examined the relationships between auditory or visual thresholds and symptoms, cognition, and functioning variables in the pHCP sample. No relationships were observed in either auditory or visual thresholds with symptoms measured by the SANS, SAPS, or BPRS (all *FDR-p*’s>.24; See **Table 2**). Additionally, no relationships were observed in auditory or visual thresholds with social or role function (all *FDR-p*’s>.24; See **Table 2**). Next we examined the relationship between auditory and visual thresholds and global cognitive scores measured by the BACS across the PSY, REL, and CON groups (controlling for age and gender). Auditory thresholds showed a strong relationship with global cognition across all groups (*t*=-4.83 *FDR-p*=.00002; *df=*121; See **Table 2A** and **Figure 2A**), where lower thresholds corresponded to higher global cognition scores. This was shown to be the case within the PSY (*r*=-.49; *p*=.00001; *df*=72; See **Figure 2B**), REL *(r*=-.37; *p*=.02; *df*=38), but not the CON group (though the direction of the association was the same; *r*=-.35; *p*=.26; *df*=10; here, the small sample size for CON in the pHCP group may limit our ability to form a strong conclusion). Visual thresholds also showed a relationship with global cognition across groups (*t*=-3.2 *FDR*-*p*=.01; *df=*121; See **Table 2A** and **Figure 2C**), again with lower thresholds corresponding to higher global cognition scores. The PSY (*r*=-.48; *p*=.00001; *df*=72; See **Figure 2D**) and REL groups (*r*=-.34; *p*=.03; *df*=38) showed this relationship when considered alone, while the CON group did not (*r*=-.1; *p*=.76; *df*=10). The regression models predicting global cognition remained significant for both auditory and verbal thresholds across groups when controlling for psychiatric symptoms measured by the BPRS (*p*’s<.002). We also confirmed that no effects on auditory threshold, visual threshold, or global cognition were being driven by a relationship with medication dosage in subjects receiving antipsychotic medication (all correlation *p*’s >.07). These findings point to a connection between impaired sensory discrimination and poorer cognitive functioning in people with psychosis, even when controlling for psychiatric symptoms.

**Table 2:** Relationships with clinical, cognitive, and functioning variables.

A) Outcome Measure	Sound Sweeps t-value (df)	p-value (FDR-corrected)	Visual Sweeps t-value (df)	p-value (FDR-corrected)
SANS	-0.12 (4,70)	0.84	1.76 (4,70)	0.24
SAPS	-0.53 (4,70)	0.72	1.24 (4,70)	0.44
BPRS	1.01 (4,121)	0.48	0.11 (4,121)	0.92
BAC Global Score	-4.83 (4,121)	0.00002	-3.20 (4,121)	0.01
GAF Role	-1.79 (4,117)	0.24	0.14 (4,117)	0.92
GAF Social	-1.43 (4,117)	0.32	-0.11 (4,117)	0.92
**B) BAC Sub-Scale**	**Auditory t-value (df)**	**p-value**	**Visual t-value (df)**	**p-value**
Motor Speed	-3.96 (4,121)	0.0001	-0.24 (4,121)	0.81
Verbal Memory	-2.54 (4,121)	0.01	-1.89 (4,121)	0.06
Working Memory	-2.9 (4,121)	0.004	-2.1 (4,121)	0.04
Verbal Fluency	-2.07 (4,121)	0.04	-0.43 (4,121)	0.67
Processing Speed	-4.31 (4,121)	0.00003	-2.08 (4,121)	0.04
Problem Solving	-3.36 (4,121)	0.001	-3.07 (4,121)	0.003

Relationships with Clinical, Cognitive, and Functioning Variables Across the Psychosis, Relative, and Control Groups. (A) Sound and Visual Sweep thresholds predicting symptoms, cognition, and functioning were modeled controlling for age and gender across groups (except for the SANS and SAPS measures which were modeled in the psychosis group alone). Significant effects survived a False-Discovery Rate (FDR) correction. (B) Sound and Visual Sweep thresholds predicting BAC Sub-scales (controlling for age and gender) were performed post-hoc, therefore the p-values are uncorrected. SANS, Scale for the Assessment of Negative Symptoms; SAPS, Scale for the Assessment of Positive Symptoms; BPRS, Brief Psychiatric Rating Scale; BAC, Brief Assessment of Cognition; GAF Role, Global Assessment of Functioning: Role; GAF Social, Global Assessment of Functioning: Social.

We followed these analyses by performing post-hoc tests examining whether the relationships between auditory and visual thresholds and global cognition were driven by specific BACS sub-tests. Lower auditory thresholds showed a significant relationship across groups with all BACS sub-domains including higher verbal memory, working memory, motor speed, verbal fluency, processing speed, and problem solving (See **Table 2B**). Lower visual thresholds showed a significant relationship with higher verbal memory, working memory, processing speed, and problem solving (See **Table 2B**).

Last, we examined the relationship between Auditory and Visual thresholds in the pHCP group. Across groups, auditory and visual sensory thresholds were found to be strongly correlated with one another (*r*=.44; *p*=1x10^-7^; *df*=131), driven by a strong relationship in the PSY group (*r*=.55; *p*=2x10^-7^; *df*=74), a statistical trend in the REL group (*r*=.28 *p*=.06; *df*=42), and no relationship in the CON group (*r*=.16; *p*=.60; *df*=11).

## Discussion

Deficits in sensory processing in both the auditory and visual domains may reflect the pathophysiology underlying psychosis and partially account for perceptual distortions and cognitive impairment noted in people with psychotic disorders. Sound and Visual Sweep paradigms are brief and standardized means for assessing sensory deficits and are easily deployed at relatively low cost on a large scale. These tasks provide indices of sensitivity to auditory and visual frequency modulation (i.e., measuring the briefest stimulus that can be reliably discriminated). Results of the present study provide evidence that auditory sensory thresholds differ among individuals with psychosis and healthy controls, with first-degree biological relatives of individuals with psychosis showing similar results to psychosis patients, but not statistically different from controls. Impaired auditory discrimination thresholds in this paradigm may serve as a psychosis endophenotype, where individuals with genetic liability for psychosis demonstrate a moderate deficit. Consistent with previous work (36), individuals with psychosis and their biological realtives who had worse auditory discrimination thresholds had worse global cognition, indicating that auditory processing deficits are related to downstream disruptions in various aspects of cognition, including memory, attention, processing speed, and problem solving. These findings were found to be significant even when controlling for psychiatric symptoms, and is consistent with recent findings demonstrating that deficits in early auditory processing in schizophrenia reflect cognitive impairments, but may not be related to positive or negative symptoms (46). Together, these findings suggest that early sensory processing disruptions may be more reflective of cognitive disruptions than psychotic symptomatology.

Similar patterns were observed in visual sensory thresholds on the Visual Sweeps task, where individuals with psychosis and their biological relatives did not differ from one another, though in the visual domain the biological relatives showed the strongest differences from the healthy control group. This suggests that visual discrimination thresholds may also possibly represent a psychosis endophenotype, though post-hoc tests revealed no significant between-group differences. Generally, worse visual discrimination thresholds in the psychosis patients and relatives were related to worse global cognition, and like auditory thresholds were associated with disruptions in memory, attention, processing speed, and problem solving. Notably, sensory thresholds in both modalities appeared to be similar across psychiatric diagnoses, suggesting that these sensory processing deficits affect a psychosis dimension more broadly.

A trend toward auditory threshold deficits in biological relatives in the present study is broadly consistent with previous electrophysiological findings that have demonstrated sensory disruptions in biological relatives of psychosis patients. Deficits in auditory MMN were found in first-degree relatives of patients with schizophrenia (47), while auditory P300 deficits have appeared in relatives of both schizophrenia and bipolar disorder patients (48–50). Not surprisingly, these electrophysiological deficits appear consistent with behavioral findings in patients and their relatives that affect higher-order auditory processes such as verbal working memory (51–53). However, key differences in auditory processing may also distinguish schizophrenia and bipolar illnesses, as well as their respective genetic liabilities. In a dichotic listening task, schizophrenia subjects and their relatives showed early (N100) auditory encoding deficits, while the bipolar subjects and their relatives did not, though both patient groups showed impaired modulation of N100 (54). This may reflect subtle differences in the role of early auditory processing with regard to pathophysiological and genetic risk markers of psychosis.

Similar electrophysiologic deficits have been observed in early visual processing in schizophrenia patients and their relatives (55), and appear to be heritable in schizophrenia and bipolar disorder (56). While the results from the Visual Sweeps task suggested that relatives had higher visual sensory thresholds (similar to that of the psychosis patients), literature examining visual surround suppression and visual motion integration across psychosis subjects and their relatives suggests a pattern of impairment associated with the clinical conditions themselves. In studies of schizophrenia, bipolar disorder, their respective relatives, and controls, surround suppression and contour detection were both found to be weaker in schizophrenia, relatively less weak in bipolar disorder, and functionally spared in both relative groups (57, 58). This suggests that gain control is impaired in psychotic illnesses, but is less likely to reflect genetic liability. A similar conclusion was drawn from a study examining visual motion integration, where patients with schizophrenia were shown to have elevated motion detection thresholds while relatives and bipolar subjects did not (59). However, select visual backward masking paradigms have yielded evidence of early perceptual abnormalities associated with genetic liability for schizophrenia (60).

In the current study, auditory and visual sensory processing thresholds were strongly correlated with one another, suggesting that perceptual dysfunction across sensory domains arrives from a common mechanism. Despite dysfunction that appears specific to the auditory and visual systems respectively (61, 62), n-methyl-D-asparate-type (NMDA) glutamate receptor dysfunction may be a common disrupted neurotransmitter pathway accounting for generalized sensory impairments (1, 63). Based on work showing impairments in both auditory and visual perception in response to NMDA blocking agents (64–66), we may speculate a model wherein NMDA receptor hypofunction impacts γ-amino-butyric-acid (GABA) interneurons that have direct influence on cortical oscillatory timing and affect attention and working memory (67). Relatedly, these GABA neurons may alter mesocortical dopamine pathways that impact aspects of sensory functioning and could potentially account for psychotic symptomatology (67). Further research will be required to understand the direct impact of NMDA dysfunction and its influence on auditory and visual sensory thresholds.

In the present study, Sound Sweeps was found to be most strongly related to the BACS processing speed subtest (Digit-Symbol Coding), which has been hypothesized to underlie the core generalizable cognitive deficit in psychosis (68–70). Thus, sweep paradigms could tap perceptual functions central to the general cognitive impairment noted in psychotic disorders such as schizophrenia. Given that neither the Sound nor Visual Sweep tasks rely on a linguistic component, they may serve as culturally unbiased perceptual assessment tools that are sensitive to global impairments. Additionally, either task is completed in three or four minutes, can be deployed remotely, and is understood by most participants without a proctor or additional instructions. As performance on these tasks can change over time and track cognitive improvements in real time (33), the sweep paradigm may be ideal for ecological momentary assessment of sensory processes related to global cognitive functioning.

A major limitation of the current study was that the control group was drawn from three separate samples. This resulted in uneven groups comparing healthy controls to psychosis probands and their relatives. Relatedly, the M-Turk Visual Sweeps group was collected remotely, so there may be subtle differences in this sample compared to other controls, though they did not statistically differ from the controls collected in person. We also note that there were only N=13 controls who were included in analyses examining the relationships between sensory thresholds and symptoms, cognition, or functioning. This limited the interpretability of these findings, as we were underpowered to confirm whether the relationship between impaired sensory thresholds and cognition is specific to psychosis subjects and their relatives, as opposed to characterizing a broader phenomenon unrelated to psychopathology.

Another limitation is that the relatives group was not limited to ‘unaffected’ relatives, such that some relatives had a psychiatric diagnosis or may have had a history of subsyndromal psychosis-like experiences. This may have contributed to higher observed thresholds in the relatives, though the results for both auditory and visual thresholds were unchanged after removing relatives with a previous psychosis diagnosis (N = 2). Further research on unaffected relatives will be important for clarifying the endophenotypic nature of this behavioral marker. Finally, while the overall sample size was modest, the delivery of the auditory and visual stimuli were not as precisely controlled as they may have been in a traditional perceptual psychophysics laboratory, and the reliability of these tasks remains to be fully tested. This limits the generalizability of findings, but did allow us to test these tools in a naturalistic setting. Further study will be necessary not only to validate auditory and visual sensory thresholds as a valid endophenotype, but also further establish their reliability and relationship to cognitive dysfunction.

Overall, the results of the current study demonstrate that sensory thresholds may represent endophenotypes present in individuals with genetic liability for a psychotic disorder. Crucially, impaired sensory thresholds were found to relate to general cognitive dysfunction across groups. Thus, these measures of early perceptual processing, which can be quickly obtained using computerized paradigms, are strongly predictive of downstream cognitive impairment.

## Data Availability Statement

The datasets generated for this study are available on request to the corresponding author.

## Ethics Statement

The studies involving human participants were reviewed and approved by University of Minnesota Institutional Review Board. The patients/participants provided their written informed consent to participate in this study.

## Author Contributions

IR was responsible for statistical analysis, data interpretation, drafted the manuscript, and oversaw data collection. M-PS assisted in statistical analysis and data interpretation. BB assisted in data interpretation and provided control data. MF, SV, and SS conceptualized and oversaw data collection. All authors contributed to the article and approved the submitted version.

## Funding

This study was supported by the National Institute of Mental Health under Award Numbers U01MH108150 (PI: SS), K01MH117451 (PI: IR), The Wells Family Foundation (PI: SV), and Posit Science Inc. internal funds. The funder Posit Science Inc. was not involved in the study design, collection, analysis, interpretation of data, the writing of this article or the decision to submit it for publication. The content is solely the responsibility of the authors and does not necessarily represent the official views of the funding agencies.

## Conflict of Interest

BB is Senior Scientist at Posit Science, a company that produces cognitive training and assessment software. Sound and Visual Sweep assessment tools in this study were provided for research purposes free of charge by Posit Science.

The remaining authors declare that the research was conducted in the absence of any commercial or financial relationships that could be construed as a potential conflict of interest.

## References

[1] Javitt DC Freedman R Sensory processing dysfunction in the personal experience and neuronal machinery of schizophrenia. Am J Psychiatry (2015) 172(1):17–31. 10.1176/appi.ajp.2014.13121691 25553496 PMC4501403

[2] Carter O Bennett D Nash T Arnold S Brown L Cai RY Sensory integration deficits support a dimensional view of psychosis and are not limited to schizophrenia. Transl Psychiatry (2017) 7(5):e1118. 10.1038/tp.2017.69 28485725 PMC5534945

[3] Uhlhaas PJ Singer W Abnormal neural oscillations and synchrony in schizophrenia. Nat Rev Neurosci (2010) 11(2):100–13. 10.1038/nrn2774 20087360

[4] Williams LE Light GA Braff DL Ramachandran VS Reduced multisensory integration in patients with schizophrenia on a target detection task. Neuropsychologia (2010) 48(10):3128–36. 10.1016/j.neuropsychologia.2010.06.028 PMC2915827 20600181

[5] McLachlan NM Phillips DS Rossell SL Wilson SJ Auditory processing and hallucinations in schizophrenia. Schizophr Res (2013) 150(2-3):380–5. 10.1016/j.schres.2013.08.039 24054462

[6] Dondé C Mondino M Leitman DI Javitt DC Suaud-Chagny M-F D’Amato T Are basic auditory processes involved in source-monitoring deficits in patients with schizophrenia? Schizophr Res (2019) 210:135–42. 10.1016/j.schres.2019.05.034 31176535

[7] Hemsley DR The development of a cognitive model of schizophrenia: placing it in context. Neurosci Biobehav Rev (2005) 29(6):977–88. 10.1016/j.neubiorev.2004.12.008 15964074

[8] Erickson MA Ruffle A Gold JM A Meta-Analysis of Mismatch Negativity in Schizophrenia: From Clinical Risk to Disease Specificity and Progression. Biol Psychiatry (2016) 79(12):980–7. 10.1016/j.biopsych.2015.08.025 PMC4775447 26444073

[9] Ford JM Mathalon DH Marsh L Faustman WO Harris D Hoff AL P300 amplitude is related to clinical state in severely and moderately ill patients with schizophrenia. Biol Psychiatry (1999) 46(1):94–101. 10.1016/S0006-3223(98)00290-X 10394478

[10] Ford JM Mathalon DH Kalba S Marsh L Pfefferbaum A N1 and P300 abnormalities in patients with schizophrenia, epilepsy, and epilepsy with schizophrenialike features. Biol Psychiatry (2001) 49(10):848–60. 10.1016/S0006-3223(00)01051-9 11343681

[11] Butler PD Javitt DC Early-stage visual processing deficits in schizophrenia. Curr Opin Psychiatry (2005) 18(2):151–7. 10.1097/00001504-200503000-00008 PMC1994776 16639168

[12] Butler PD Martinez A Foxe JJ Kim D Zemon V Silipo G Subcortical visual dysfunction in schizophrenia drives secondary cortical impairments. Brain (2007) 130(Pt 2):417–30. 10.1093/brain/awl233 PMC2072909 16984902

[13] Lalor EC De Sanctis P Krakowski MI Foxe JJ Visual sensory processing deficits in schizophrenia: is there anything to the magnocellular account? Schizophr Res (2012) 139(1-3):246–52. 10.1016/j.schres.2012.05.022 PMC3393820 22704644

[14] Calderone DJ Martinez A Zemon V Hoptman MJ Hu G Watkins JE Comparison of psychophysical, electrophysiological, and fMRI assessment of visual contrast responses in patients with schizophrenia. Neuroimage (2013) 67:153–62. 10.1016/j.neuroimage.2012.11.019 PMC3544989 23194815

[15] VanMeerten NJ Dubke RE Stanwyck JJ Kang SS Sponheim SR Abnormal early brain responses during visual search are evident in schizophrenia but not bipolar affective disorder. Schizophr Res (2016) 170(1):102–8. 10.1016/j.schres.2015.11.007 26603466

[16] Lynn PA Kang SS Sponheim SR Impaired retrieval processes evident during visual working memory in schizophrenia. Schizophr Res Cogn (2016) Sep 5:47–55. 10.1016/j.scog.2016.07.002 PMC5514301 28740817

[17] Hermens DF Chitty KM Kaur M Mismatch negativity in bipolar disorder: A neurophysiological biomarker of intermediate effect? Schizophr Res (2018) 191:132–9. 10.1016/j.schres.2017.04.026 28450056

[18] Maekawa T Katsuki S Kishimoto J Onitsuka T Ogata K Yamasaki T Altered visual information processing systems in bipolar disorder: evidence from visual MMN and P3. Front Hum Neurosci (2013) 7:403. 10.3389/fnhum.2013.00403 23898256 PMC3724050

[19] Yeap S Kelly SP Reilly RB Thakore JH Foxe JJ Visual sensory processing deficits in patients with bipolar disorder revealed through high-density electrical mapping. J Psychiatry Neurosci (2009) 34(6):459–64. PMC2783437 19949722

[20] Pokorny VJ Lano TJ Schallmo M-P Olman CA Sponheim SR Reduced influence of perceptual context in schizophrenia: behavioral and neurophysiological evidence. Psychol Med (2019) 1–9. 10.1017/S0033291719003751 PMC7444089 31858929

[21] Thomas ML Green MF Hellemann G Sugar CA Tarasenko M Calkins ME Modeling Deficits From Early Auditory Information Processing to Psychosocial Functioning in Schizophrenia. JAMA Psychiatry (2017) 74(1):37–46. 10.1001/jamapsychiatry.2016.2980 27926742 PMC5453308

[22] Rassovsky Y Horan WP Lee J Sergi MJ Green MF Pathways between early visual processing and functional outcome in schizophrenia. Psychol Med (2011) 41(3):487–97. 10.1017/S0033291710001054 PMC5534526 20482936

[23] Biagianti B Fisher M Brandrett B Schlosser D Loewy R Nahum M Development and testing of a web-based battery to remotely assess cognitive health in individuals with schizophrenia. Schizophr Res (2019) 208:250–7. 10.1016/j.schres.2019.01.047 PMC6544475 30733167

[24] Fisher M Loewy R Carter C Lee A Ragland JD Niendam T Neuroplasticity-based auditory training via laptop computer improves cognition in young individuals with recent onset schizophrenia. Schizophr Bull (2015) 41(1):250–8. 10.1093/schbul/sbt232 PMC4266283 24444862

[25] Fisher M Holland C Merzenich MM Vinogradov S Using neuroplasticity-based auditory training to improve verbal memory in schizophrenia. Am J Psychiatry (2009) 166(7):805–11. 10.1176/appi.ajp.2009.08050757 PMC2720319 19448187

[26] Thomas ML Bismark AW Joshi YB Tarasenko M Treichler EBH Hochberger WC Targeted cognitive training improves auditory and verbal outcomes among treatment refractory schizophrenia patients mandated to residential care. Schizophr Res (2018) 202:378–84. 10.1016/j.schres.2018.07.025 PMC7409526 30055883

[27] Ramsay IS Fryer S Boos A Roach BJ Fisher M Loewy R Response to Targeted Cognitive Training Correlates with Change in Thalamic Volume in a Randomized Trial for Early Schizophrenia. Neuropsychopharmacology (2018) 43(3):590–7. 10.1038/npp.2017.213 PMC5770762 28895568

[28] Subramaniam K Luks TL Garrett C Chung C Fisher M Nagarajan S Intensive cognitive training in schizophrenia enhances working memory and associated prefrontal cortical efficiency in a manner that drives long-term functional gains. Neuroimage (2014) 99:281–92. 10.1016/j.neuroimage.2014.05.057 PMC4498800 24867353

[29] Ramsay IS Roach BJ Fryer S Fisher M Loewy R Ford JM Increased global cognition correlates with increased thalamo-temporal connectivity in response to targeted cognitive training for recent onset schizophrenia. Schizophr Res (2020) 218:131–137. 10.1016/j.schres.2020.01.020 32007346 PMC7299776

[30] Popov T Jordanov T Rockstroh B Elbert T Merzenich MM Miller GA Specific cognitive training normalizes auditory sensory gating in schizophrenia: a randomized trial. Biol Psychiatry (2011) 69(5):465–71. 10.1016/j.biopsych.2010.09.028 21092939

[31] Biagianti B Roach BJ Fisher M Loewy R Ford JM Vinogradov S Trait aspects of auditory mismatch negativity predict response to auditory training in individuals with early illness schizophrenia. Neuropsychiatr Electrophysiol (2017) 3:2. 10.1186/s40810-017-0024-9 28845238 PMC5568850

[32] Dale CL Brown EG Fisher M Herman AB Dowling AF Hinkley LB Auditory Cortical Plasticity Drives Training-Induced Cognitive Changes in Schizophrenia. Schizophr Bull (2016) 42(1):220–8. 10.1093/schbul/sbv087 PMC4681549 26152668

[33] Biagianti B Fisher M Neilands TB Loewy R Vinogradov S Engagement with the auditory processing system during targeted auditory cognitive training mediates changes in cognitive outcomes in individuals with schizophrenia. Neuropsychology (2016) 30(8):998–1008. 10.1037/neu0000311 27617637 PMC5088059

[34] Perez VB Miyakoshi M Makeig SD Light GA Mismatch negativity reveals plasticity in cortical dynamics after 1-hour of auditory training exercises. Int J Psychophysiol (2019) 145:40–7. 10.1016/j.ijpsycho.2019.06.003 31176741

[35] Hochberger WC Joshi YB Thomas ML Zhang W Bismark AW Treichler EBH Neurophysiologic measures of target engagement predict response to auditory-based cognitive training in treatment refractory schizophrenia. Neuropsychopharmacology (2019) 44(3):606–12. 10.1038/s41386-018-0256-9 PMC6333927 30377381

[36] Tarasenko M Perez VB Pianka ST Vinogradov S Braff DL Swerdlow NR Measuring the capacity for auditory system plasticity: An examination of performance gains during initial exposure to auditory-targeted cognitive training in schizophrenia. Schizophr Res (2016) 172(1):123–30. 10.1016/j.schres.2016.01.019 PMC5072522 26851143

[37] Light GA Swerdlow NR Braff DL Preattentive sensory processing as indexed by the MMN and P3a brain responses is associated with cognitive and psychosocial functioning in healthy adults. J Cognit Neurosci (2007) 19(10):1624–32. 10.1162/jocn.2007.19.10.1624 PMC2562660 18271737

[38] Sponheim SR McGuire KA Stanwyck JJ Neural anomalies during sustained attention in first-degree biological relatives of schizophrenia patients. Biol Psychiatry (2006) 60(3):242–52. 10.1016/j.biopsych.2005.11.017 16460700

[39] García-Pérez MA Forced-choice staircases with fixed step sizes: asymptotic and small-sample properties. Vision Res (1998) 38(12):1861–81. 10.1016/S0042-6989(97)00340-4 9797963

[40] Andreasen NC Grove WM Evaluation of positive and negative symptoms in schizophrenia. Psychiatrie & Psychobiologie (1986) 1(2):108–21.

[41] Andreasen NC The Scale for the Assessment of Negative Symptoms (SANS): conceptual and theoretical foundations. Br J Psychiatry Suppl (1989) 155(S7):49–58. 10.1192/S0007125000291496 2695141

[42] Lukoff D Nuechterlein KH Ventura J Manual for the expanded brief psychiatric rating scale. Schizophr Bull (1986) 12:594–602. 10.1093/schbul/12.4.578

[43] Keefe RSE Goldberg TE Harvey PD Gold JM Poe MP Coughenour L The Brief Assessment of Cognition in Schizophrenia: reliability, sensitivity, and comparison with a standard neurocognitive battery. Schizophr Res (2004) 68(2-3):283–97. 10.1037/t38021-000 15099610

[44] Keefe RSE Harvey PD Goldberg TE Gold JM Walker TM Kennel C Norms and standardization of the Brief Assessment of Cognition in Schizophrenia (BACS). Schizophr Res (2008) 102(1-3):108–15. 10.1016/j.schres.2008.03.024 18495435

[45] Cornblatt BA Auther AM Niendam T Smith CW Zinberg J Bearden CE Preliminary findings for two new measures of social and role functioning in the prodromal phase of schizophrenia. Schizophr Bull (2007) 33(3):688–702. 10.1093/schbul/sbm029 17440198 PMC2526147

[46] Dondé C Martínez A Kantrowitz JT Silipo G Dias EC Patel GH Bimodal distribution of tone-matching deficits indicates discrete pathophysiological entities within the syndrome of schizophrenia. Transl Psychiatry (2019) 9(1):221. 10.1038/s41398-019-0557-8 31492832 PMC6731304

[47] Jessen F Fries T Kucharski C Nishimura T Hoenig K Maier W Amplitude reduction of the mismatch negativity in first-degree relatives of patients with schizophrenia. Neurosci Lett (2001) 309(3):185–8. 10.1016/S0304-3940(01)02072-9 11514072

[48] Schulze KK Hall M-H McDonald C Marshall N Walshe M Murray RM Auditory P300 in patients with bipolar disorder and their unaffected relatives. Bipolar Disord (2008) 10(3):377–86. 10.1111/j.1399-5618.2007.00527.x 18402626

[49] Hall M-H Schulze K Rijsdijk F Kalidindi S McDonald C Bramon E Are auditory P300 and duration MMN heritable and putative endophenotypes of psychotic bipolar disorder? A Maudsley Bipolar Twin and Family Study. Psychol Med (2009) 39(8):1277–87. 10.1017/S0033291709005261 19250581

[50] Winterer G Egan MF Raedler T Sanchez C Jones DW Coppola R P300 and genetic risk for schizophrenia. Arch Gen Psychiatry (2003) 60(11):1158–67. 10.1001/archpsyc.60.11.1158 14609891

[51] Bora E Vahip S Akdeniz F Ilerisoy H Aldemir E Alkan M Executive and verbal working memory dysfunction in first-degree relatives of patients with bipolar disorder. Psychiatry Res (2008) 161(3):318–24. 10.1016/j.psychres.2007.09.002 18977035

[52] Horan WP Braff DL Nuechterlein KH Sugar CA Cadenhead KS Calkins ME Verbal working memory impairments in individuals with schizophrenia and their first-degree relatives: Findings from the Consortium on the Genetics of Schizophrenia. Schizophr Res (2008) 103(1):218–28. 10.1016/j.schres.2008.02.014 PMC2529172 18406578

[53] Conklin HM Curtis CE Katsanis J Iacono WG Verbal working memory impairment in schizophrenia patients and their first-degree relatives: evidence from the digit span task. Am J Psychiatry (2000) 157(2):275–7. 10.1176/appi.ajp.157.2.275 10671401

[54] Force RB Venables NC Sponheim SR An auditory processing abnormality specific to liability for schizophrenia. Schizophr Res (2008) 103(1-3):298–310. 10.1016/j.schres.2008.04.038 18571375 PMC3816098

[55] Yeap S Kelly SP Sehatpour P Magno E Javitt DC Garavan H Early visual sensory deficits as endophenotypes for schizophrenia: high-density electrical mapping in clinically unaffected first-degree relatives. Arch Gen Psychiatry (2006) 63(11):1180–8. 10.1001/archpsyc.63.11.1180 17088498

[56] Bestelmeyer PEG Phillips LH Crombie C Benson P St Clair D The P300 as a possible endophenotype for schizophrenia and bipolar disorder: Evidence from twin and patient studies. Psychiatry Res (2009) 169(3):212–9. 10.1016/j.psychres.2008.06.035 19748132

[57] Schallmo M-P Sponheim SR Olman CA Reduced contextual effects on visual contrast perception in schizophrenia and bipolar affective disorder. Psychol Med (2015) 45(16):3527–37. 10.1017/S0033291715001439 PMC4624017 26315020

[58] Schallmo M-P Sponheim SR Olman CA Abnormal contextual modulation of visual contour detection in patients with schizophrenia. PloS One (2013) 8(6):e68090. 10.1371/journal.pone.0068090 23922637 PMC3688981

[59] Chen Y Bidwell LC Holzman PS Visual motion integration in schizophrenia patients, their first-degree relatives, and patients with bipolar disorder. Schizophr Res (2005) 74(2-3):271–81. 10.1016/j.schres.2004.04.002 15722006

[60] Sponheim SR Sass SM Noukki AL Hegeman BM Fragile early visual percepts mark genetic liability specific to schizophrenia. Schizophr Bull (2013) 39(4):839–47. 10.1093/schbul/sbs041 PMC3686444 22446567

[61] Martínez A Hillyard SA Bickel S Dias EC Butler PD Javitt DC Consequences of magnocellular dysfunction on processing attended information in schizophrenia. Cereb Cortex (2012) 22(6):1282–93. 10.1093/cercor/bhr195 PMC3357176 21840846

[62] Javitt DC Sweet RA Auditory dysfunction in schizophrenia: integrating clinical and basic features. Nat Rev Neurosci (2015) 16(9):535–50. 10.1038/nrn4002 PMC4692466 26289573

[63] Kantrowitz JT Javitt DC N-methyl-d-aspartate (NMDA) receptor dysfunction or dysregulation: the final common pathway on the road to schizophrenia? Brain Res Bull (2010) 83(3-4):108–21. 10.1016/j.brainresbull.2010.04.006 PMC2941541 20417696

[64] Koychev I William Deakin JF El-Deredy W Haenschel C Effects of Acute Ketamine Infusion on Visual Working Memory: Event-Related Potentials. Biol Psychiatry Cognit Neurosci Neuroimaging (2017) 2(3):253–62. 10.1016/j.bpsc.2016.09.008 29528296

[65] Schmidt A Bachmann R Kometer M Csomor PA Stephan KE Seifritz E Mismatch negativity encoding of prediction errors predicts S-ketamine-induced cognitive impairments. Neuropsychopharmacology (2012) 37(4):865–75. 10.1038/npp.2011.261 PMC3280661 22030715

[66] Rosburg T Kreitschmann-Andermahr I The effects of ketamine on the mismatch negativity (MMN) in humans–a meta-analysis. Clin Neurophysiol (2016) 127(2):1387–94. 10.1016/j.clinph.2015.10.062 26699665

[67] Balu DT The NMDA Receptor and Schizophrenia: From Pathophysiology to Treatment. Adv Pharmacol (2016) 76:351–82. 10.1016/bs.apha.2016.01.006 PMC5518924 27288082

[68] Dickinson D Ramsey ME Gold JM Overlooking the obvious: a meta-analytic comparison of digit symbol coding tasks and other cognitive measures in schizophrenia. Arch Gen Psychiatry (2007) 64(5):532–42. 10.1001/archpsyc.64.5.532 17485605

[69] González-Blanch C Rodríguez-Sánchez JM Pérez-Iglesias R Pardo-García G Martínez-García O Vázquez-Barquero JL First-episode schizophrenia patients neuropsychologically within the normal limits: Evidence of deterioration in speed of processing. Schizophr Res (2010) 119(1):18–26. 10.1016/j.schres.2010.02.1072 20335007

[70] Rodríguez-Sánchez JM Crespo-Facorro B González-Blanch C Perez-Iglesias R Vázquez-Barquero JL PAFIP Group Study Cognitive dysfunction in first-episode psychosis: the processing speed hypothesis. Br J Psychiatry Suppl (2007) 51:s107–10. 10.1192/bjp.191.51.s107 18055925

